# Impact of Polyethylene-Glycol-Induced Water Potential on Methane Yield and Microbial Consortium Dynamics in the Anaerobic Degradation of Glucose

**DOI:** 10.3390/bioengineering11050433

**Published:** 2024-04-27

**Authors:** Jin Yeo, Yong-Woo Jeon

**Affiliations:** 1 Biogas Research Center, Hankyong National University, Anseong 17579, Republic of Korea; jin90405@naver.com; 2 Environmental Technology Division, Korea Testing Laboratory, Seoul 08389, Republic of Korea

**Keywords:** water potential, polyethylene glycol, cation inhibition, anaerobic digestion, methane production, toxic effect

## Abstract

This study investigated the relationship between water potential (Ψ) and the cation-induced inhibition of methane production in anaerobic digesters. The Ψ around methanogens was manipulated using polyethylene glycol (PEG) in a batch anaerobic reactor, ranging from −0.92 to −5.10 MPa. The ultimate methane potential (B_u_) decreased significantly from 0.293 to 0.002 Nm^3^ kg^−1^-VS_added_ as Ψ decreased. When Ψ lowered from −0.92 MPa to −1.48 MPa, the community distribution of acetoclastic *Methanosarcina* decreased from 59.62% to 40.44%, while those of hydrogenotrophic *Methanoculleus* and *Methanobacterium* increased from 17.70% and 1.30% to 36.30% and 18.07%, respectively. These results mirrored changes observed in methanogenic communities affected by cation inhibition with KCl. Our findings strongly indicate that the inhibitory effect of cations on methane production may stem more from the water stress induced by cations than from their direct toxic effects. This study highlights the importance of considering Ψ dynamics in understanding cation-mediated inhibition in anaerobic digesters, providing insights into optimizing microbial processes for enhanced methane production from organic substrates.

## 1. Introduction

Water potential (Ψ) represents the chemical potential of moisture within a system, influenced by a combination of physical and chemical factors. Total Ψ generally consists of osmotic (Ψ_o_), gravitational (Ψ_g_), matric (Ψ_m_), and pressure potential (Ψ_p_). In physicochemical systems, the value of Ψ is assigned as 0 in pure water, with negative values indicating Ψ in relation to pure water [1]. In microbial reaction systems, Ψ is predominantly dictated by Ψ_o_, a colligative property influenced by salt and organic acid concentrations. Elevated salt content results in diminished Ψ, leading to water stress, and inhibiting the physiological activity of microorganisms and higher plant tissue due to low Ψ [2,3]. Ψ serves as a physicochemical indicator of moisture movement in soil and water environments, as well as the water availability of plants or microorganisms. Under low Ψ conditions, microorganisms experience water stress due to reduced water availability. The consequential decrease in intracellular water content disrupts various enzymatic reactions, significantly hindering microbial physiological activity [4,5,6,7,8]. Microorganisms manage Ψ, allowing stable moisture uptake as Ψ spontaneously moves from high to low, ensuring optimal conditions for physiological activities and survival.

Anaerobic digesters represent complex colloidal systems comprising particulate and dissolved organic matter, diverse ionic substances, and microorganisms [9,10]. The process of anaerobic digestion involves the production of methane from organic matter. The decomposition of high-molecular organic matter into low-molecular organic matter occurs through hydrolysis, acidogenesis, and acetogenesis reaction stages, with the production of various organic and amino acids during this process [11]. Additionally, the ionization of minerals during the decomposition of organic matter can influence the Ψ of the anaerobic digester. Notably, a substantial amount of salt, as found in food waste, induces a rapid decrease in Ψ within the anaerobic digester. While numerous studies have explored the impact of Ψ on water uptake by higher plants in soil and methane production from soil organic matter [12,13,14], research on the variation of Ψ in anaerobic digesters and the effect of Ψ on the efficiency of anaerobic digestion is limited.

Due to South Korea’s unique dietary culture, characterized by the consumption of large amounts of salt, food waste in the country has been found to have a high salt content of approximately 3% based on dry matter weight [15]. Consequently, concerns about operational difficulties are commonly raised in anaerobic digestion facilities that process food waste, given the high salt content of the raw materials. Despite these concerns, there is a lack of comprehensive research on the diversity of salt species and their interactions with anaerobic microorganisms, which hampers the development of effective operational strategies to manage salt effects in anaerobic digesters. While previous research has investigated cations such as sodium (Na^+^), potassium (K^+^), calcium (Ca^2+^), and magnesium ions (Mg^2+^) in anaerobic digesters to understand their impact on Ψ and anaerobic microorganisms [16,17], the focus has primarily been on the inhibitory effects of individual cationic species. Significantly, research addressing the interaction between anaerobic microorganisms and cations remains inadequate. Progress in understanding these interactions is crucial for enhancing the operational efficiency of anaerobic digesters processing high-salt-content food waste.

Because Ψ represents the colligative properties of the solution affected by factors such as salt and organic acid concentration, it can be used as an indicator for interpreting the overall interaction between the efficiency of the anaerobic digester and salt effects. Yeo et al. [18] analyzed Ψ in 20 South Korean anaerobic digestion facilities, reporting an average Ψ of approximately −1.23 MPa (±0.65). Introducing Ψ to assess the impact of salt on digester efficiency, a water potential inhibition assay in a batch anaerobic reactor, where Ψ was manipulated by varying K^+^ concentration, indicated methane production inhibition below Ψ −1.65 MPa (a K^+^ concentration of 11.69 g/L). According to this report, the inhibitory effect of a high K^+^ concentration on methane production may stem from the induced decrease in Ψ, challenging the prevailing notion of a direct inhibition effect of K^+^ ions on methanogenesis. However, thus far, in studies related to cation inhibition by K^+^, many researchers have determined that the optimal concentration range of K^+^ for anaerobic digesters was 3000 to 28,000 mg/L [18,19,20,21,22,23]. According to another report, when the appropriate concentration range of K^+^ was exceeded, methane production was inhibited via cation (K^+^) inhibition. However, K^+^ is the principal intracellular cation in bacteria and eukaryotic cells. The accumulation of K^+^ in bacteria can occur via different transport systems that vary in kinetics, energy coupling, and regulation [24]. Accordingly, the assertion that inhibition by K^+^ in anaerobic digesters stems directly from the toxic effect of K^+^ ions remains contentious. Generally, elevated cation concentrations can lead to the concomitant induction of low Ψ in an anaerobic digester, and the cation inhibition effect is accompanied by another inhibitory effect caused by low Ψ. Thus, it is unclear whether the inhibition of methane production by cations is a direct toxic effect of cations or an inhibitory effect of low Ψ derived from cations.

Despite numerous investigations highlighting the inhibitory impact of cations on anaerobic digestion, there is limited research exploring the influence of Ψ on the anaerobic production of methane from organic substrates [25,26,27]. Notably, the concurrent occurrence of cation-induced inhibition and alterations in Ψ poses challenges in elucidating the overarching inhibitory effects of Ψ factors on anaerobic digestion. In this study, we sought to discern the specific impact of water potential on anaerobic methane production, mitigating the confounding effects of cation inhibition. To achieve this, polyethylene glycol (PEG; (C_2_H_4_O)_n_H_2_O) was employed to regulate Ψ in a batch anaerobic reactor [28]. Subsequently, an assessment of methane production efficiency from glucose was conducted under controlled Ψ conditions induced by PEG. Furthermore, we utilized next-generation sequencing (NGS) technology to analyze shifts in microbial communities attributable to variations in Ψ.

## 2. Materials and Methods

### 2.1. Theoretical Methane Potential (B_th_)

The stoichiometric calculation of the theoretical methane potential (B_th_) was performed using Boyle’s equation, which was based on the results obtained from the elemental analysis of the samples (Equations (1) and (2)) [29].

(1) $$\begin{array}{c} C_{a}H_{b}O_{c}N_{d}S_{e}+\left(a-\frac{b}{4}-\frac{c}{2}+\frac{3d}{4}+\frac{e}{2}\right)H_{2}O\to \left(\frac{a}{2}+\frac{b}{8}-\frac{c}{4}-\frac{3d}{8}-\frac{e}{4}\right)CH_{4}+\left(\frac{a}{2}-\frac{b}{8}+\frac{c}{4}+\frac{3d}{8}+\frac{e}{4}\right)CO_{2}+dNH_{3}+eH_{2}S \end{array}$$

(2) $$\begin{array}{c} B_{th}\left(Nm^{3}{kg}^{-1}-{VS}_{added}\right)=22.4\times \left[\frac{\left(4a+b-2c-3d-2e)/8\right)}{12a+b+16c+14d+32e}\right] \end{array}$$

### 2.2. Methane Production Potential Assay

#### 2.2.1. Anaerobic Decomposition Characteristics of PEG

To investigate the impact of PEG degradation on the effectiveness of Ψ inhibition assays, batch anaerobic reactors were set up for the degradation assay using PEG 4000, as outlined in Table 1. The Ψ levels in the batch anaerobic reactor were adjusted to −0.88, −1.47, −3.00, −4.00, and −5.11 MPa using PEG 4000, and the decomposition characteristics of PEG used in Ψ adjustment were assessed.

#### 2.2.2. Water Potential (Ψ) Inhibition Assay

The batch anaerobic reactors employed in the Ψ inhibition assay were prepared according to the specifications outlined in Table 2. D-glucose served as the substrate, with a consistent substrate to inoculum ratio of 0.5 (g-VS_substrate_ g^−1^–VS_inoculum_) across all anaerobic batch reactors, as detailed in Table 2. To conduct the Ψ inhibition assay, the Ψ for each batch anaerobic reactor was precisely adjusted to −1.48, −2.97, −4.01, and −5.10 MPa utilizing a PEG 4000 solution.

#### 2.2.3. Ultimate Methane Potential (B_u_)

Evaluation of the ultimate methane potential (Bu) was conducted using the biochemical methane potential (BMP) assay [30]. A batch-type anaerobic reactor was operated under mesophilic conditions at 38 °C. The anaerobic inoculum used in this study was obtained from a farm-scale anaerobic digester located in Icheon City, South Korea. Detailed information regarding the chemical properties of the inoculum is provided in Table 3.

The inoculum used in the BMP assay was incubated under mesophilic conditions at 38 °C for two weeks to eliminate any remaining biodegradable components. The headspace of the serum bottles was purged with N_2_ gas and then sealed with a butyl rubber stopper. The Control group, which did not receive PEG addition, served as the baseline treatment. Furthermore, a blank test was conducted using a batch anaerobic reactor containing 70 mL of inoculum, with glucose omitted to measure biogas produced solely by the inoculum. Sample, Control, and blank anaerobic batch reactors were operated in triplicate to ensure robust experimental consistency.

Each anaerobic batch reactor underwent an incubation period lasting up to 78 days within a convection incubator. To optimize fermentation, manual mixing procedures were performed daily throughout the incubation period. Determination of the ultimate methane potential relied on the volatile solid (VS) content. For precision, the ultimate methane potentials of the samples were adjusted using the blank value and standardized under standard temperature and pressure (STP) conditions at 0 °C and 1 atm, respectively. The modified Gompertz model (Equation (3)) [31] was employed to track the cumulative methane production progress, facilitating data optimization using the same equation.

(3) $$\begin{array}{c} M=P\times \exp\left[-exp\frac{R_{m}\times e}{P}\left(\lambda -t\right)+1\right] \end{array}$$

where M is the cumulative methane production (mL), t is the anaerobic fermentation time (days), P is the final methane production (mL), e is the exp (1), R_m_ is the maximum rate of methane production (mL day^−1^), and λ is the lag growth phase time (days). The cumulative methane production curves determined in the BMP assay were optimized using SigmaPlot (SigmaPlot Version 12.5, Systat Software Inc., Chicago, IL, USA) using the modified Gompertz model.

### 2.3. Microbial Community Analysis

#### 2.3.1. DNA Extraction and Quantification

Extraction of DNA was performed using a DNeasyPowerSoil Kit (Qiagen, Hilden, Germany) according to the manufacturer’s instructions. Quant-IT PicoGreen (Invitrogen) was used for the quantification of the extracted DNA [18].

#### 2.3.2. Library Construction and Sequencing

The preparation of sequencing libraries followed the Illumina 16S Metagenomic Sequencing Library protocols, specifically tailored for amplifying the archaeal region. Initially, 5 ng of genomic DNA (gDNA) underwent amplification via polymerase chain reaction (PCR). The reaction included a 5× reaction buffer, 1 mM dNTP mix, 500 nM of each universal forward and reverse PCR primer, and Herculase II fusion DNA polymerase (Agilent Technologies, Santa Clara, CA, USA). The PCR cycle conditions consisted of a 3 min heat activation at 95 °C, followed by 25 cycles of 30 s at 95 °C, 30 s at 63 °C, and 30 s at 72 °C, concluding with a 5 min final extension at 72 °C [18]. The universal primer pair, inclusive of Illumina adapter overhang sequences, utilized for the first amplification were as follows: 787-F (5′-TCGTCGGCAGCGTCAGATGTGTATAAGAGACAGATTAGATACCCSBGTAGTCC-3′) and 1059-R (5′-GTCTCGTGGGCT CGGAGATGTGTATAAGAGACAGGCCATGCACCWCCTCT-3′). Following the first PCR, the product underwent purification using AMPure beads (Agen-court Bioscience, Beverly, MA, USA). For the construction of the final library, 2 µL of the purified first PCR product underwent amplification with PCR incorporating the index using NexteraXT Indexed Primer. The cycle conditions for the second PCR mirrored those of the first PCR, except for a reduced cycle number of 10 [18]. The resulting PCR product was again purified using AMPure beads. Quantification of the final purified product was conducted through qPCR following the qPCR Quantification Protocol Guide (KAPA Library Quantification kits for Illumina Sequencing platforms), and its quality was assessed using TapeStation D1000 ScreenTape (Agilent Technologies, Waldbronn, Germany). Paired-end sequencing (2 × 300 bp) was subsequently carried out by Macrogen, utilizing the MiSeq™ platform (Illumina, San Diego, CA, USA).

### 2.4. Analysis

#### 2.4.1. Water Potential (Ψ) Analysis

The determination of Ψ was carried out using the WP4C instrument (METER Group, Inc., Pullman, WA, USA), which operates on the chilled mirror dew point principle. In this method, air within the chamber containing the sample is directed over the cooling mirror, leading to condensation on the mirror’s surface. The Ψ of a liquid sample is determined by establishing a correlation between the sample’s Ψ reading and the saturation vapor pressure of air in equilibrium with the sample. This correlation is defined by Equation (4) [32].

(4) $$\begin{array}{c} \Psi =\frac{RT}{M_{w}}\ln\frac{e_{s}\left(T_{d}\right)}{e_{s}\left(T_{s}\right)} \end{array}$$

where Ψ is the water potential (MPa), $e_{s}(T_{d})$ is the saturation vapor pressure of the air at dew point temperature, $e_{s}(T_{s})$ is the saturation vapor pressure at the sample temperature, R is the gas constant, 8.31 J mol^−1^ K^−1^, T is the Kelvin temperature of the sample, and $M_{w}$ is the molecular mass of water.

#### 2.4.2. Chemical Analysis

The characterization of physicochemical properties in the biochemical methane potential (BMP) assay was conducted following established protocols [33]. A comprehensive analysis, including pH, alkalinity, total solids (TSs), volatile solids (VSs), fixed solids (FSs), chemical oxygen demand (COD_Cr_), soluble chemical oxygen demand (SCOD_Cr_), total Kjeldahl nitrogen (TKN), ammonium nitrogen (NH_4_^+^-N), and total volatile fatty acids (TVFAs), was performed to assess both the chemical characteristics and organic composition. Specifically, volatile fatty acids (VFAs) were quantified using a gas chromatograph (GC2010, Shimadzu Scientific Instruments, Inc., Columbia, MD, USA) equipped with a flame ionization detector and an automatic sampler. For the determination of gas concentrations, a gas chromatograph (Clarus 680, PerkinElmer, Inc., Waltham, MA, USA) equipped with a thermal conductivity detector and a HayeSepQ packed column (CRS, Inc., Louisville, KY, USA) was employed. Helium served as the carrier gas at a flow rate of 5 mL/min during column operation. The injector, oven, and detector temperatures were set at 150 °C, 90 °C, and 150 °C, respectively [34].

#### 2.4.3. Statistical Analysis

The tables display the mean values and corresponding standard deviations derived from the experimental data. Statistical analyses of the results were conducted using R Studio (R Ver. 4.1.3, R Foundation for Statistical Computing, Vienna, Austria). Utilizing analysis of variance (ANOVA) in conjunction with Duncan’s multiple range tests facilitated the identification of statistically significant differences (*p* < 0.05) among the mean values of the different treatment groups.

## 3. Results and Discussion

### 3.1. Anaerobic Degradation Assay

**Figure 1:**
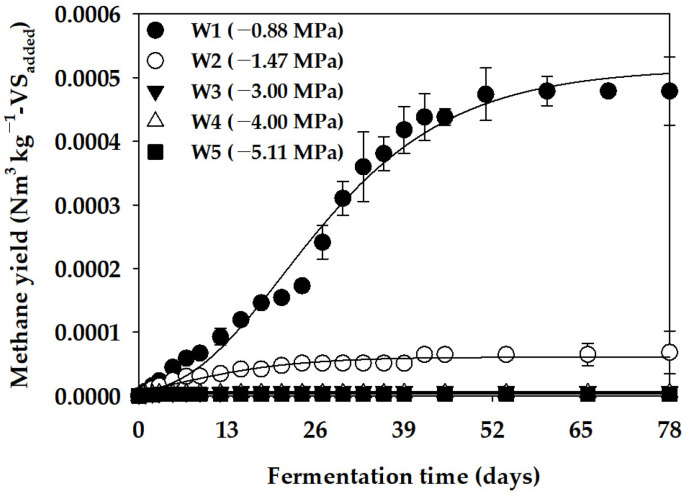
Cumulative methane yield curve for PEG 4000 (vertical bars represent standard deviations).

In this study, we aimed to determine the effect of the Ψ factor on the anaerobic production of methane while discounting the influence of cation inhibition. We utilized PEG 4000 to adjust Ψ in the batch anaerobic reactor. The anaerobic degradation characteristics of PEG 4000 were analyzed to assess its impact on Ψ adjustment in the inhibition assay. Figure 1 illustrates the cumulative methane production curve obtained from the anaerobic degradation assay, while Table 4 presents the parameters of the modified Gompertz model derived by optimizing the cumulative methane production curve. In operating the batch anaerobic reactor for 78 days under Ψ conditions of −0.88 MPa (W1), we observed a B_u_ of PEG 4000 at 0.001 Nm^3^ kg^−1^-VS_added_, with an anaerobic degradation ratio (VS_r_) of 0.08% at that time. Furthermore, a minimal anaerobic decomposition of PEG 4000 was observed under Ψ conditions of −1.47 MPa (W2) or lower. These results indicate that anaerobic microorganisms can utilize PEG 4000 as a carbon source for methane production, albeit with low efficiency. Huang et al. [35] investigated the microbiological decomposition rate of PEG with various molecular weights (600, 6000, and 20,000) under aerobic and anaerobic conditions. They reported a lower decomposition rate under anaerobic conditions, particularly noting a further reduction in the anaerobic degradation rate of PEG when glucose was added as an external carbon source. Dwyer and Tiedje [36] also noted that PEG 4000, being a high-molecular-weight substance, decomposes at a relatively slow rate under anaerobic conditions. Consequently, our evaluation suggests that the effect of the anaerobic degradation of PEG in the inhibition assay using D-glucose was relatively low.

### 3.2. Water Potential (Ψ) Inhibition Assay

**Figure 2:**
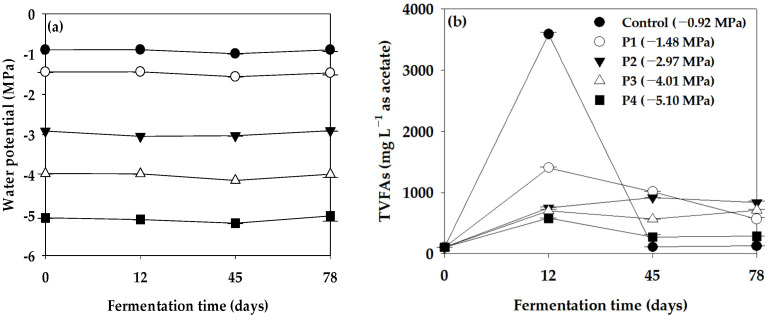
Variation in (**a**) water potential (Ψ) and (**b**) TVFAs in the batch anaerobic reactor during the Ψ inhibition assay (vertical bars represent standard deviations).

The measurement of Ψ and TVFAs in the anaerobic reactor was conducted at 0, 12, 45, and 78 days to assess the variability of Ψ during its operational period (Figure 2). Initially, in the Control (−0.92 MPa), the TVFA content measured 108, 3588, 113, and 127 mg L^−1^ at days 0, 12, 45, and 78, respectively. Notably, TVFAs exhibited a significant surge to 3588 mg L^−1^ by the 12th day of reactor operation. In the anaerobic reactor with controlled Ψ, the TVFA content at −1.48 MPa (P1) was 106, 1406, 1015, and 567 mg L^−1^, while at −2.97 MPa (P2), it measured 106, 747, 919, and 837 mg L^−1^ at 0, 12, 45, and 78 days, respectively. Moreover, at −4.01 MPa (P3), the TVFA content was 105, 703, 565, and 713 mg L^−1^, and at −5.10 MPa (P4), it was 106, 580, 273, and 286 mg L^−1^ at the same respective time points. Consequently, lower Ψ in the anaerobic reactor correlated with decreased TVFA concentration, indicating an inhibitory effect on the anaerobic microorganisms involved in acidogenesis and acetogenesis, where TVFAs are produced within the anaerobic reactor.

Additionally, the Ψ of the Control (−0.92 MPa) ranged from −0.99 to −0.89 MPa, and the Ψ conditions remained stable throughout the operation period of the anaerobic reactor, despite a significant increase in the content of TVFAs at 12 days of operation. There were no significant changes in the Ψ conditions. Furthermore, during the operation period of the anaerobic reactor, the Ψ conditions for P1 (−1.56 to −1.44 MPa), P2 (−3.04 to −2.90 MPa), P3 (−4.14 to −3.97 MPa), and P4 (−5.20 to −5.02 MPa) remained stable.

**Figure 3:**
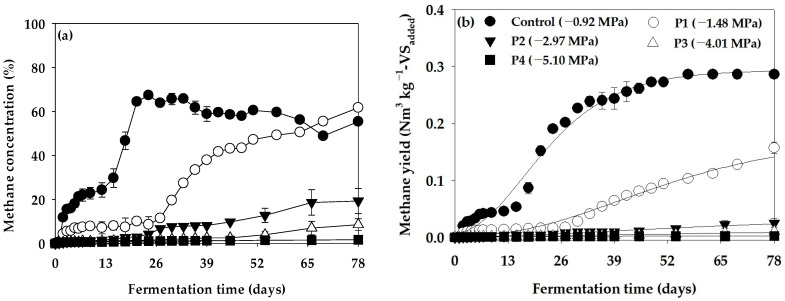
Variation in (**a**) methane concentration and (**b**) cumulative methane yield curves in the water potential (Ψ) inhibition assay (vertical bars represent standard deviations).

The methane concentration curve and cumulative methane production curve obtained using the Ψ inhibition assay are shown in Figure 3, and the parameters derived by optimizing the cumulative methane production curve using the modified Gompertz model are shown in Table 5. In the Ψ inhibition assay, the B_u_ for Control (−0.92 MPa) was 0.293 Nm^3^ kg^−1^-VS_added_, and it decreased to 0.176, 0.034, 0.014, and 0.002 Nm^3^ kg^−1^-VS_added_ as Ψ of the anaerobic reactor decreased to −1.48 (P1), −2.97 (P2), −4.01 (P3), and −5.10 MPa (P4), respectively. R_m_ was 4.34 mL day^−1^ in the Control (−0.92 MPa), 1.28 mL day^−1^ in −1.48 MPa (P1), 0.19 mL day^−1^ in −2.97 MPa (P2), 0.06 mL day^−1^ in −4.01 MPa (P3), and 0.03 mL day^−1^ in −5.10 MPa (P4), showing a rapidly slowing trend as Ψ decreased. In particular, λ was 4.95 days in −0.92 MPa (Control), but significantly extended to 15.96 days in −1.48 MPa (P1). However, methane production showed minimal progression at −2.97 MPa (P2), −4.01 MPa (P3), and −5.10 MPa (P4). Thus, the value of λ derived from the modified Gompertz model was not valid. In this study, the efficiency of methane production showed a significant decrease as the Ψ of the anaerobic reactor decreased. Since the toxic effect of PEG on organisms is minimal [37], the effect of anaerobic digestion inhibition by Ψ induced by PEG 4000 can typically be evaluated as a direct effect of the Ψ.

In this study, significant differences were observed in methane production characteristics when comparing the Control (−0.92 MPa) in the Ψ inhibition assay to decreasing Ψ levels in the anaerobic reactor. Specifically, at −1.48 MPa (P1), methane production exhibited a gradual increase after approximately 26 days of batch anaerobic reactor operation. By day 78, the methane potential reached 0.176 Nm^3^ kg^−1^-VS_added_, indicating the restoration of microbial activity in the anaerobic reactor. Thus, under Ψ conditions of −1.48 MPa (P1), although methane production was initially inhibited, the subsequent recovery of anaerobic reactor activity occurred with a sufficient acclimatization time. Yeo et al. [18] reported similar findings in their Ψ inhibition assay using KCl, noting a rapid decrease in methane yield and production rate at the onset of anaerobic reactor operation at a −2.85 MPa Ψ level. However, the gradual recovery of anaerobic microorganism activity was observed with an adequate acclimatization time. In contrast, in our study, where Ψ was adjusted using PEG 4000, the restoration of anaerobic reactor activity through acclimatization was not evident at −2.97 MPa (P2), a Ψ level comparable to −2.85 MPa [18]. Anaerobic reactor activity restoration through acclimatization was only confirmed at the highest Ψ level of −1.48 MPa (P1). Furthermore, when K^+^ was utilized as a Ψ adjuster [18], anaerobic reactor activity restoration by acclimatization was only confirmed at the highest Ψ level of −1.48 MPa (P1). In addition, when K^+^ was used as a Ψ adjuster [18], methane production was inhibited by 34.85% at −3.91 MPa compared to the Control. In contrast, our study showed a significant suppression of methane production by 95.22% compared to the Control at −4.01 MPa (P3), a Ψ level similar to −3.91 MPa. Generally, anaerobic microorganisms adapt to low Ψ environments by accumulating K^+^ and compatible solutes within cells. Several studies have reported on the survival of methanogens in salt-stress environments [38,39,40,41,42]. Additionally, low Ψ induced by solutes permeable to the cell wall, such as cations, may cause protoplasm separation, with cells reverting to their original state in a hypotonic solution environment [43,44]. However, studies have indicated that Ψ inhibition by polymers impermeable to the cell wall, such as PEG, can exert intense pressure on cells, leading to cell wall collapse [44,45,46]. Consequently, compared to Ψ induced by cations, the effect of PEG-induced Ψ on cell activity recovery in anaerobic microorganisms can be fatal, potentially resulting in cell wall collapse.

### 3.3. Microbial Community

**Figure 4:**
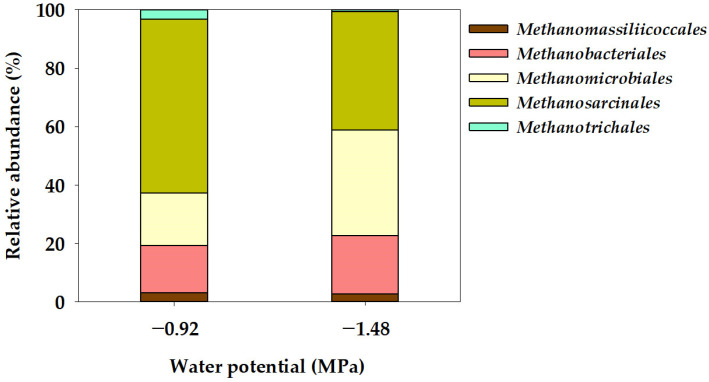
Order-level taxonomic composition of the archaeal community in the water potential (Ψ) inhibition assay.

**Figure 5:**
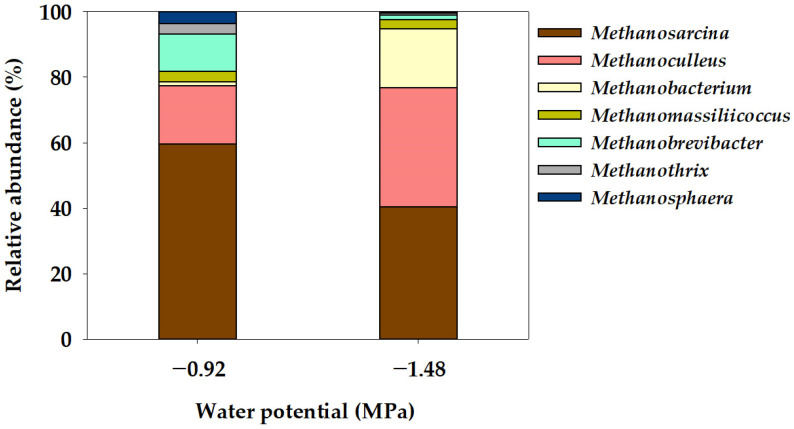
Genus-level taxonomic composition of the archaeal community in the water potential (Ψ) inhibition assay.

In the Ψ inhibition assay, the microbial community characteristics of methanogens at the order and genus level were determined using the NGS technique after operating a batch anaerobic reactor for 78 days, as illustrated in Figure 4 and Figure 5. Methanogenic archaea are slow-growing microorganisms, and methane is produced by maintaining a syntrophic mechanism with various microorganisms involved in acetogenesis [47]. In this study, the methanogen community comprised five orders: *Methanomassiliicoccales*, *Methanobacteriales*, *Methanomicrobiales*, *Methanosarcinales*, and *Methanotrichales* (Figure 4). *Methanosarcinales* exhibited the most extensive community distribution, accounting for 59.52% in −0.92 MPa (Control), yet this proportion decreased to 40.44% in −1.48 MPa (P1). Additionally, *Methanotrichales* accounted for 3.26% in −0.92 MPa (Control) and decreased to 0.63% in −1.48 MPa (P1). However, the community distribution of *Methanobacteriales* increased from 16.27% in −0.92 MPa (Control) to 19.84% in −1.48 MPa (P1), while the distribution of *Methanomicrobiales* also significantly increased from 17.85% in −0.92 MPa (Control) to 36.32% in −1.48 MPa (P1). The mechanism for methane production in methanogenic bacteria can be categorized into four pathways: CO_2_ reduction, acetoclastic, methyl reduction, and methylotrophic pathways [48]. The *Methanosarcinales* order can utilize all four pathways for methane production [49,50]. The *Methanosarcina* genus, belonging to the *Methanosarcinales* order and known as an acetoclastic methanogen [51], exhibited a community distribution of 59.62% in −0.92 MPa (Control), which significantly decreased to 40.44% in −1.48 MPa (P1). The community distribution of the *Methanothrix* genus decreased from 3.27% in −0.92 MPa (Control) to 0.63% in −1.48 MPa (P1). The *Methanotrichales* order, to which the *Methanothrix* genus belongs, utilizes the acetoclastic pathway, converting acetate into CH_4_ and CO_2_ [52]. However, the community distribution of *Methanobacterium* and *Methanoculleus* genera significantly increased from 1.30% and 17.70% in −0.92 MPa (Control) to 18.07% and 36.30%, respectively. In general, in the *Methanomicrobiales* and *Methanobacteriales* orders, CO_2_ is reduced to CH_4_ using H_2_ as an electron donor through the CO_2_ reduction pathway [50,53]. The genera *Methanoculleus*, *Methanobacterium*, and *Methanobrevibacter*, belonging to these orders, are known as hydrogenotrophic methanogens [52,54]. Yeo et al. [18] reported that the results of the Ψ inhibition assay induced by KCl showed a decrease in the community distribution of acetoclastic methanogens and an increase in the community distribution of hydrogenotrophic methanogens at a low Ψ of −3.91 MPa. Additionally, under low Ψ conditions, a significantly increased dominance of hydrogenotrophic methanogens (*Methanobacterium* and *Methanoculleus* genera) was observed, suggesting that hydrogenotrophic methanogens can be advantageous for acclimation under low Ψ conditions. Meanwhile, the genus *Methanomassiliicoccus*, belonging to the *Methanomassiliicoccales* order, is known to utilize the methyl reduction pathway [55]. The community distribution of the *Methanomassiliicoccus* genus showed a slight decrease from 3.11% in the Control (−0.92 MPa) to 2.78% in P1 (−1.48 MPa).

In this study, the methanogen community characteristics of P2 (−2.97 MPa), P3 (−4.01 MPa), and P4 (−5.10 MPa) could not be identified due to a lack of appropriate DNA extraction. Here, PEG 4000 was used to control Ψ, and Ψ at −2.97 MPa (P2) occurred at a PEG 4000 concentration of approximately 30% or more. Therefore, it is assumed that the extraction of DNA was impeded by the excessive use of PEG 4000 to induce the desired Ψ conditions. According to one report, interference with DNA extraction by PEG can cause cytorrhysis via osmotic pressure applied to cells by polymer solutes that are impermeable to the cell wall, such as PEG [28]. Additionally, some reports suggest that cells respond to DNA damage in a hypertonic solution environment, and sudden increases in osmotic pressure result in apoptosis [56]. In particular, Evilevitch et al. [57] reported that DNA extraction was entirely inhibited by high osmotic pressure at a PEG concentration of 29%. In this study, PEG 4000 was used to prevent the inhibitory effect of cations in the performance of the Ψ inhibition assay. However, an excessive concentration of PEG (used to induce very low Ψ conditions) led to the inhibition of the analysis of the methanogen population in the anaerobic reactor. These experimental cases should be considered cautiously in future research on Ψ using PEG.

## 4. Conclusions

The primary objective of this study was to determine the isolated impact of the Ψ factor on the efficacy of anaerobic digestion inhibition. At the Ψ condition of −0.92 MPa (Control), the specific methane production (B_u_) amounted to 0.293 Nm^3^ kg^−1^-VS_added_. However, a notable reduction was observed at −1.48 MPa (P1), where B_u_ decreased significantly to 0.176 Nm^3^ kg^−1^-VS_added_. Additionally, B_u_ showed a significant decrease as the Ψ of the anaerobic reactor decreased, and under the lowest Ψ condition (−5.10 MPa; P4), it was 0.002 Nm^3^ kg^−1^-VS_added_, indicating that methane production was almost completely suppressed. R_m_, which represents the rate of methane production during the process of anaerobic digestion, also showed the same trend as B_u_. Therefore, the results demonstrated that a decrease in Ψ in the anaerobic reactor had a direct inhibitory effect on the conversion of organic matter to methane. The analysis of the methanogen community distribution characteristics within the anaerobic reactor revealed that under the Ψ condition of −0.92 MPa (Control), the community distribution of *Methanosarcina* genus, recognized for employing the acetoclastic pathway, constituted 59.62%. In contrast, this distribution decreased to 40.44% under the lowest Ψ condition of −1.48 MPa (P1). Moreover, the community distribution of the *Methanoculleus* and *Methanobacterium* genera, associated with the hydrogenotrophic pathway, was 17.70% and 1.30%, respectively, at −0.92 MPa (Control), but exhibited an increase to 36.30% and 18.07%, respectively, under the reduced Ψ condition of −1.48 MPa (P1). These findings suggest that the cation-induced suppression of methane production may be due to the inhibitory effect of Ψ induced by cations, rather than a direct impact of cations themselves. Ψ is influenced by various substances present in the anaerobic digester, including not only cations, but also anions, organic acids, amino acids, and proteins. Further research on the interaction of these substances is needed for a more specific understanding of Ψ’s effect on anaerobic digestion.

## Figures and Tables

**Table 1:** Experimental scheme for the anaerobic degradation assay using polyethylene glycol (PEG).

Parameters		Blank	Treatments				
			W1	W2	W3	W4	W5
Inoculum (mL)		70	70	70	70	70	70
Substrate ^1^ solution	PEG conc. ^2^ (%)	-	16.25	22.75	31.85	35.75	39.00
	Volume (mL)	-	130	130	130	130	130
D.W. ^3^ volume (mL)		130	-	-	-	-	-
Operation volume (mL)		200	200	200	200	200	200
Ψ ^4^ (MPa)		−0.36 (0.03) ^5^	−0.88 (0.00)	−1.47 (0.02)	−3.00 (0.06)	−4.00 (0.11)	−5.11 (0.05)

^1^ PEG 4000 used as substrate. ^2^ PEG 4000 concentration in operation volume. ^3^ Distilled water. ^4^ Water potential for the batch anaerobic reactor. ^5^ Standard deviation.

**Table 2:** Experimental scheme for the water potential (Ψ) inhibition assay.

Parameters		Blank	Control	Treatments			
				P1	P2	P3	P4
Inoculum (mL)		70	70	70	70	70	70
Substrate ^1^ (g)		-	0.52	0.52	0.52	0.52	0.52
S/I ratio ^2^ (-)		-	0.5	0.5	0.5	0.5	0.5
W.P. ^3^ adjusting solution	PEG ^4^ conc. (%)	-	-	22.75	31.85	35.75	39.00
	Volume (mL)	-	-	130	130	130	130
D.W. ^5^ volume (mL)		130	130	-	-	-	-
Operation volume (mL)		200	200	200	200	200	200
Ψ ^6^ (MPa)		−0.94 (0.05) ^7^	−0.92 (0.05)	−1.48 (0.06)	−2.97 (0.07)	−4.01 (0.08)	−5.10 (0.08)

^1^ D-Glucose used as substrate. ^2^ Substrate to inoculum ratio (g-VS_substrate_ g^−1^–VS_inoculum_). ^3^ Water potential. ^4^ PEG 4000 concentration in operation volume. ^5^ Distilled water. ^6^ Water potential for the batch anaerobic reactor. ^7^ Standard deviation.

**Table 3:** Chemical composition of inoculum used in the anaerobic degradation assay using PEG and the water potential (Ψ) inhibition assay.

Parameters	pH	TS ^1^	vs. ^2^	TCOD ^3^	SCOD ^4^	TKN ^5^	NH_4_^+^-N ^6^	Alkalinity	TVFAs ^7^
	(-)	(mg L^−1^)	(mg L^−1^)	(mg L^−1^)	(mg L^−1^)	(mg L^−1^)	(mg L^−1^)	(mg L^−1^ as CaCO_3_)	(mg L^−1^ as Acetate)
Inoculum	7.96	30,044	14,767	21,050	6012	2201	1314	6389	113

^1^ Total solid. ^2^ Volatile solid. ^3^ Total chemical oxygen demand. ^4^ Soluble chemical oxygen demand. ^5^ Total Kjeldahl nitrogen. ^6^ Ammonium nitrogen. ^7^ Total volatile fatty acids.

**Table 4:** Ultimate methane yield and the parameters calculated using the modified Gompertz model for the anaerobic degradation assay using PEG 4000.

Parameters		Ψ Treatments ^1^				
		W1	W2	W3	W4	W5
Ψ ^2^ (MPa)		−0.88	−1.47	−3.00	−4.00	−5.11
VS_r_ ^3^ (%)		0.08	0.01	0.00	0.00	0.00
Model parameters	B_u_ ^4^ (Nm^3^ kg^−1^-VS_added_)	0.001	0.000	0.000	0.000	0.000
	P ^5^ (mL)	16.0	3.0	0.4	0.3	0.2
	R_m_ ^6^ (mL day^−1^)	0.43	0.11	0.04	0.13	0.08
	λ ^7^ (days)	7.34	i.v. ^8^	i.v.	i.v.	i.v.
	R^2^	0.973	0.855	0.875	0.939	0.733

^1^ Water potential was adjusted using PEG 4000 solution in the anaerobic degradation assay. ^2^ Water potential. ^3^ Ratio of anaerobic degradation (B_u_/B_th_×100; B_th_ of PEG 4000 = 0.633 Nm^3^ kg^−1^-VS_added_). ^4^ Ultimate methane potential. ^5^ Maximum methane production. ^6^ Maximum methane production rate. ^7^ Lag growth phase time. ^8^ Invalid value.

**Table 5:** Ultimate methane yield and the parameters calculated using the modified Gompertz model for the water potential (Ψ) inhibition assay.

Parameters		Control	Treatments ^1^			
			P1	P2	P3	P4
Ψ ^2^ (MPa)		−0.92	−1.48	−2.97	−4.01	−5.10
VS_r_ ^3^ (%)		78.64 a ^8^	47.31 b	8.98 c	3.65 d	0.62 e
Model parameters	B_u_ ^4^ (Nm^3^ kg^−1^-VS_added_)	0.293 a	0.176 b	0.034 c	0.014 d	0.002 e
	P ^5^ (mL)	141 a	85 b	16 c	7 d	1 e
	R_m_ ^6^ (mL day^−1^)	4.34 a	1.28 b	0.19 c	0.06 d	0.03 e
	λ ^7^ (days)	4.95 c	15.96 a	i.v. ^9^	i.v.	i.v.
	R^2^	0.981	0.980	0.903	0.875	0.915

^1^ Water potential was adjusted using PEG 4000 solution in the water potential inhibition assay. ^2^ Water potential. ^3^ Ratio of anaerobic degradation (B_u_/B_th_ × 100; B_th_ of D-glucose = 0.373 Nm^3^ kg^−1^-VS_added_). ^4^ Methane potential. ^5^ Maximum methane production. ^6^ Maximum methane production rate. ^7^ Lag growth phase time. ^8^ Different letters (a–e) indicate significant differences between treatments (DMRT; Duncan’s multiple range test, *p* < 0.05). ^9^ Invalid value.

## Data Availability

The original contributions presented in the study are included in the article; further inquiries can be directed to the corresponding author.
